# Prevalence of Tinea Capitis among Children in Osogbo, Nigeria, and the Associated Risk Factors

**DOI:** 10.3390/diseases7010013

**Published:** 2019-01-27

**Authors:** Yemisi O. Adesiji, Busayo F. Omolade, Isaac A. Aderibigbe, Oluwabunmi V. Ogungbe, Olusegun A. Adefioye, Samuel A. Adedokun, Margaret A. Adekanle, Richard O. Ojedele

**Affiliations:** 1 Department of Medical Microbiology and Parasitology, College of Health Sciences, Faculty of Basic Medical Sciences, Ladoke Akintola University of Technology, Osogbo PMB 4400, Nigeria; omolade.busayo@yahoo.com (B.F.O.); oaadefioye@lautech.edu.ng (O.A.A.); saadedokun27@lautech.edu.ng (S.A.A.); alabaadekanle@yahoo.com (M.A.A.); richardjune121988@gmail.com (R.O.O.); 2 Department of Dermatology and Venereology, Obafemi Awolowo University, Ile-Ife A234, Nigeria; ajibolajoko@yahoo.com; 3 Department of Epidemiology and Biostatistics, School of Public Health. Jackson State University, Jackson, MS 39213, USA

**Keywords:** tinea capitis, dermatophytes, school children, Nigeria

## Abstract

Tinea capitis is a fungi infection of the scalp that disproportionately affects children in rural and underserved communities in Nigeria. A case-control study was conducted to identify the causative agents and factors that predispose school pupils to tinea capitis in two selected government-owned public primary schools in Osogbo, Southwestern Nigeria. A total of 230 participants were included in the study: 115 cases and 115 controls. Head scrapings were collected from pupils with suspected T. capitis lesions, viewed under Potassium Hydroxide smear microscopy and cultured in Sabouraud’s Dextrose Agar (SDA) for characteristic fungal elements. A total of 105 (91%) samples were successfully cultured, of which 56% (59/105) were from male pupils. *Tricophyton rubrum* (34%), *Tricophyton mentagrophyte* (31%) and *Microsporum canis* (18%) were the most prevalent organisms. Other dermatophytes obtained include *Microsporum nanuum* (3%)*, Epidermophyton floccusum* (6%), *Tricophyton verucosum* (1%), and *Microsporum gypseum* (8%). Pupils between the ages of 4 and 7 years had the highest distribution (67%) followed by those between the ages of 8 and 11 years (39%). Playing with animals, the sharing of combs and not bathing with soap were significantly associated with tinea capitis infection (*P* < 0.05) in each case. This study showed a high prevalence of tinea capitis caused by the identified dermatophytes in the area.

## 1. Introduction

Globally, fungal infections are very significant diseases. Their prevalence ranges from one country to another. The high burden of serious fungal infections has been demonstrated in some parts of the world [1], including Nigeria. Every year, more than 11.8% of the Nigerian population is estimated to suffer from a serious fungal infection. Approximately 960,000 of those who are affected suffer considerable mortality [1]. 

The clinical presentation of tinea capitis varies due to a range of factors, such as the type of organism causing the infection, the inflammatory response of the host, and the type of hair infection. This is particularly common in zoophilic infections [2]. Tinea capitis may begin as a non-inflammatory black dot on the scalp, before hair shafts around the area begin to weaken and hairs break off. Eventually, a well-defined area of hair loss is produced. Other kinds of infection patterns include the non-inflammatory seborrheic dermatitis and the inflammatory type, kerion celsi [3]. In some carriers, tinea capitis may also be asymptomatic. 

The incidence of tinea capitis among children is greater in developing countries. This has been attributed to inadequacies of improved social, economic, healthcare and hygiene practices; this includes poor living conditions, children’s interaction patterns, poor sanitation, housing congestion, limited water supply, and poor health-seeking behavior. There is also a higher susceptibility among children who have pets, wet skin conditions, skin injuries or abrasions, and those who use public showers, are barefoot, and share hairbrushes or unwashed clothing with other people [4]. The prevalence of tinea capitis is equally high among pre-pubertal children [3]. It is seen more frequently among primary school children between 5 and 10 years of age and is more prevalent among poor families who have an average of five or more children [2,5]. Dermatophytes are commonly found in tropical and subtropical regions, which is the climate in most regions of the continent of Africa. This explains the etiological observation that this group of fungi are known to grow best in warm and humid environments [6]. Nigeria, located in West Africa, has a tropical climate, and most of the regions in the country have a wet humid climate; this renders the area a supportive environment for a high prevalence of dermatophytosis. 

Some studies have highlighted the prevalence of dermatophytes in some states in Nigeria. A study conducted at Lagos University Teaching Hospital (LUTH) in Southwestern Nigeria revealed that 41% of the 395 patients visiting the state hospital were infected with dermatophytoses; they found *M. canis* to be the most prevalent species [7]. In a similar study on dermatophytes among primary school children in Abia state, Southeastern Nigeria, *T. mentagrophytes* was the predominant dermatophyte found [8]. In Ogun State, Southwestern Nigeria, most of the dermatophyte identified were anthropophilic except *M. canis* which was the only zoophilic dermatophyte identified in the study [9]. Studies have attributed the strengthening of the animal–human transmission route of this fungi infection to the keeping of free-range animals as pets, with little or no veterinary checks [6].

Tinea capitis is a highly contagious fungi infection and compliance with the treatment, which is required to last for several weeks, may be difficult to achieve [2,3]. With the very high prevalence of tinea capitis among school children in Osun state, Southwestern Nigeria [10], we conducted a study to identify the causative agents and factors that predispose school pupils to tinea capitis in two selected government-owned public primary schools in Osogbo, Nigeria.

## 2. Materials and Methods

### 2.1. Study Area and Study Population

The study was carried out in two selected government-owned public primary schools in Osogbo, Osun State, Nigeria, among preadolescent children of ages 4–15 years. Students whose parents/guardians had signed the permission form were included in the study. They were interviewed and screened. The information obtained from them included their personal hygiene practices (whether they bathe with soap, how often they bathe, how often they change their uniform), whether they share combs, scarves, and hats; their playing habits, which include contact with animals or sand; the socio-economic status of their parents; their living conditions; the number of students in their classrooms. All information was obtained directly from the child (direct interview) and in some cases from the school personal profile on the child. Children who were eligible, and were between the ages of 4 and 15 years, were screened. A total of 115 students who showed visible clinical signs of dermatophyte infection, with scaling, constituted the study population while another 115 students with no sign of infection served as the control. 

### 2.2. Sample Collection and Handling

Samples were obtained by scraping the infected head area with a sterile scalpel, one for each child. The collection site was swabbed with 70% (*v/v*) ethanol before sample collection. The scrapings were collected into a sterile paper, folded in a well-labeled envelope to avoid exposure to moisture and sunlight, and also to prevent the growth of contaminants. The envelope was labeled (with the student’s name, age, sex, date of collection) and taken to the laboratory for analysis. An adequate amount of sample material was collected which ensured enough was available for all the investigations required. 

### 2.3. Detection, Isolation and Characterization of Fungal Pathogens

A portion of each sample was placed on a slide and a drop of an aqueous solution of 10% (w/v) potassium hydroxide, KOH, was added. After 5 min, the wet mount was examined under low (×10) and high (×40) power magnification for the presence of fungal elements such as arthrospores (arthroconidia, macro and/or microconidia and chlamydospores). Each of the samples was cultured on Sabouraud’s Dextrose Agar (SDA) with 0.05 mg of chloramphenicol and 0.05 mg of cycloheximide. The SDA was prepared by dissolving 65 g in one liter of distilled water. The resulting medium was autoclaved at 95 °C for 15 min and then allowed to cool to a temperature of 43 °C. Thereafter, 15–20 mL of the molten agar medium was poured into each sterilized Petri dish and allowed to solidify. 

The agar was inoculated by transferring some of the hair scales to the surface of the medium using a sterile wire loop and forceps. The plates were labeled and then incubated for three weeks at 27–30 °C, aerobically, for at least 3 weeks before being discarded as a negative result. Further identification after the growth of the dermatophytes was established; the sub-culture was also made on Sabouraud’s dextrose agar. The mycelium and spore characteristics were noted. The identification of the dermatophytes from the positive cultures was based on the colonial characteristics in pure culture and the microscopic morphology of fungi using lactophenol blue, which includes the presence of conidia (macro and micro) and the microscopic appearance of the conidia.

### 2.4. Statistical Analysis

Data obtained from this study were analyzed using SPSS Inc. (IBM version 23) (IBM Corp, Armonk, NY, USA). Significant differences between variables were determined using chi-square and *p* < 0.05 was taken as a significant value.

### 2.5. Ethical Approval

Ethical clearance was obtained from the Osun State Ministry of Education, Abeere, Osun state. Informed consent was obtained from the parents and guardians of the students of the schools before the samples were collected. All collected data were handled with confidentiality; the data were inspected, de-identified, encrypted and stored in the primary investigator’s laboratory computer. The respondents were given the right to refuse to take part in the study, and they could withdraw at any time during the study period. Participants who had scaly skin lesions, scaling and hair loss on the scalp and whose parents/guardians did not consent were excluded. However, the students without the infection served as controls.

## 3. Results

Out of the 115 head scraping samples obtained from pupils with tinea capitis lesions, 105 (91.35%) were successfully cultured, of which 56% (59/105) were males and 44% (46/105) were females. Among the pupils, *Tricophyton rubrum* (34%), *Tricophyton mentagrophyte* (31%) and *Microsporum canis* (18%) were the most prevalent dermatophytes. Other dermatophytes obtained included *Microsporum nanuum* (3%)*, Epidermophyton floccusum* (6%)*, Tricophyton verucosum* (1%) and *microsporum gypseum* (8%). The pupils between the ages of 4 and 7 years had the highest distribution (67%) followed by those between the ages of 8 and 11 years (39%). Pupils whose ages were between 12 and 15 years (3%) had the lowest distribution of dermatophytes. Playing with animals, the sharing of combs and not bathing with soap were significantly associated with tinea capitis infection (*p* < 0.05) in each case. Based on the income classification of parents, children who had tinea capitis were overwhelmingly (93%) from low-income households. The socio-demographical characteristics of the participants are presented in Table 1.

### 3.1. Prevalence of Tinea Capitis among Pupils with Clinical Symptoms

The samples from ten pupils with clinical symptoms yielded no growth during analysis, the remaining 105 pupils with clinical symptoms were positive for tinea capitis. The prevalence of tinea capitis observed among the subjects was *T. rubrum* (34%), followed by *T. mentagrophyte* (31%), *M. canis* (18%), *M. gypseum* (8%), *E. flocussum* (6%), *M. nauum* (3%), and *T. verucossum* (1%) (Table 2).

### 3.2. Risk Factors Associated with the Occurrence of Tinea Capitis among All the Participants

We observed risk factors that are known to be associated with the occurrence of tinea capitis among the participants. This was based on how often they bathed, whether they used soap for bathing, how often they changed their uniforms, whether they shared combs, caps or scarves, and their playing habits (Table 3). The chi-square test of independence was used to show if an association exists between these risk factors (independent variables) and the presence of Tinea capitis. 

#### 3.2.1. Frequency of Baths, and Use of Bath Soaps

Three quarters of the participants (*n* = 172, 75%) said that they have one bath every day. More than half of those who have baths daily had tinea capitis. We found that a significant association existed between tinea capitis and the frequency of baths; pupils who had more baths per day were less likely to have the infection *p* < 0.001 (Table 3a). Many of the pupils (59) said that they use soaps for their baths. Amongst those who did not use soap regularly, almost all of them (99%) had tinea capitis. Tinea capitis was significantly associated with neglecting the use of bath soaps χ^2^ = 10.804, df = 1. *p* < 0.001.

#### 3.2.2. How Often School Uniforms Are Changed

We asked pupils how often they changed their school uniforms. The Majority of them (58%) said that they did so once every week. Another 37% said that they change their uniforms two times every week. The infection was found to be significantly associated with how frequently pupils changed their uniforms, χ^2^ = 33.383, df = 3. *p* < 0.001. Compared to those who changed their uniforms two times every week, pupils who changed their uniforms only once a week were more likely to have tinea capitis; 23 (27%) versus 82 (61%), respectively (Table 3a).

#### 3.2.3. Hair Combs, Caps and Headscarves Sharing

**Figure 1:**
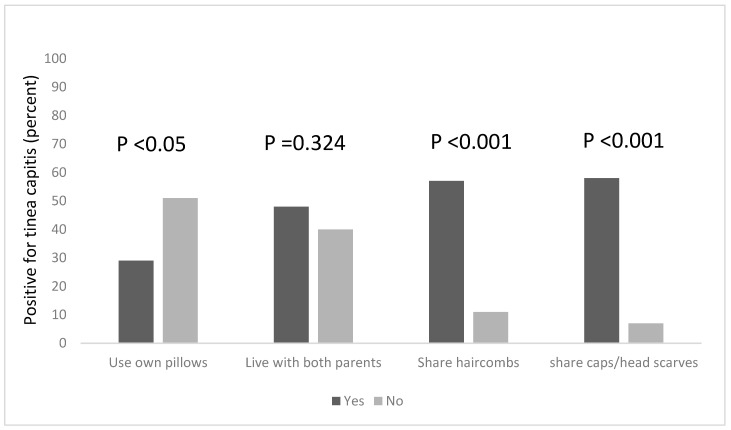
Risk factors associated with the occurrence of tinea capitis among the participants, based on the sharing of personal items and clothing.

Combs are potential fomites in the transmission of tinea capitis, and this practice is known to facilitate the transmission of tinea capitis (Figure 1). Among the pupils in this study, more than half (57%) of the pupils who shared hair combs had the infection, compared to 11% of those who did not share combs but still had the infection. This was found to be statistically significant, χ^2^ = 36.418, df = 1, *p* < 0.001. Tinea capitis was also found to be significantly associated with the sharing of caps and headscarves, χ^2^ = 42.915, df = 1, *p* < 0.001; among pupils who shared these clothing items with others, 58% of them had tinea capitis, while 7% of those who did not share caps and headscarves had the infection.

#### 3.2.4. Playing Habits

When asked whether they played with animals or sand, more than half of the pupils (56%) said they played more with sand. However, 66% of those who played more with animals had tinea capitis. We found a significant association between the playing habits of the pupils and tinea capitis, χ^2^ = 29.649, df = 1. * *p* < 0.001. The rate of the infection was higher among those who played with animals (Table 3a).

### 3.3. Risk Factors Associated with the Occurrence of Tinea Capitis among All the Participants, Based on Household Information

**Figure 2:**
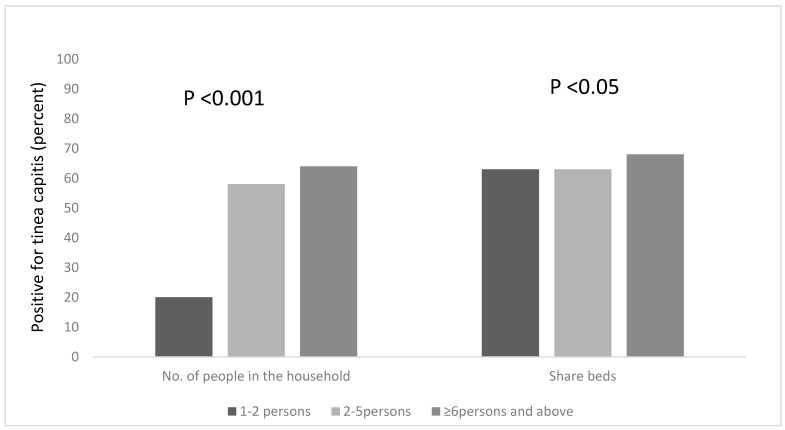
Occurrence of tinea capitis among the participants, based on the number of people in the household and the sharing of beds.

We explored other risk factors associated with the occurrence of Tinea capitis among the participants, based on household information, such as the number of people in the household, the number of people who sleep on the same bed and those who own a pillow to themselves. We found that a statistical association existed between the number of people living in a household, and being infected with tinea capitis, χ^2^ = 18.208, df = 2, *p* < 0.001. Pupils who lived in households that had six occupants or more were more likely to be infected; the majority of the pupils (65%) lived in houses with six or more occupants (Figure 2) and 64% of this group were infected with tinea capitis. The occurrence of tinea capitis among those who shared beds with ≥6 persons was about 5% more than those did so with less persons (Figure 2). Many of the participants (82%) also reported that they do not own their own pillows, and approximately 29% of those who shared pillows had tinea capitis (Figure 1).

### 3.4. Occurrence of Tinea Capitis Based on Socio-Demographical Data of the Participants

Using a chi-square test of independence, we examined the statistical association that exists between socio-demographic data and infection with tinea capitis (Table 3b). We found a significant association between tinea capitis infection and age groups, χ^2^ = 4.566, df = 2. *p* < 0.001. There was a higher prevalence of infection among pupils aged 4–7 years; 63% of those who had the infection were in this age category, followed by those aged 8–11 years (32%), and only 5% of pupils aged 12–15 years had tinea capitis. Tinea capitis was not found to be significantly associated with a pupil’s gender, although more male pupils (56%) compared to females had the infection, χ^2^ = 1.249, df = 1 *p* = 0.264. 

An overwhelming majority (93%) of the participants were from low-income households (parents earn less than #29,999 per month). However, we did not observe a statistically significant relationship between the income status of parents and infection with tinea capitis, χ^2^ = 5.151, df = 2, *p* = 0.076. 

## 4. Discussion

Tinea capitis is a highly contagious fungi infection, with a high prevalence among pre-pubertal children, and endemicity in several regions of Nigeria [1,6,11]. In this study, we isolated and identified the dermatophytes that caused tinea capitis amongst children attending two selected government-owned public primary schools in Osogbo, Nigeria. We found a high prevalence of the infection among children aged 4–7 years. The dermatophytes that were isolated, in the order most prevalent, were *T. rubrum, T. mentagrophyte*, *M. canis*, *M.gypseum*, *E.flocussum*, *M.nauum*, and *T.verucossum*. We also assessed the associations of tinea capitis with certain risk factors.

The most prevalent dermatophytes causing tinea capitis in our study setting are slightly different from those found in other studies that were also conducted in Southwestern Nigeria. A particular study in Ile-Ife, Osun state, a town in the same state where our study was carried out, isolated *Microsporum audouinii* as the most prevalent organism among the pupils who were studied [10]. We did not isolate any *Microsporum audouinii* in this study. However, the most occurring dermatophyte in our study, *T. rubrum*, has also been the most isolated organism among children with tinea capitis infection in certain parts of Northern Nigeria [12]. We suspect that *T. rubrum* may have become more prevalent in Southwestern Nigeria than originally thought.

*T. mentagrophyte*, which is the second most common dermatophyte that we found in this study, has a wider distribution in various parts of Nigeria compared to *T. rubrum*. The former has been found in various states in almost every region in the country. *T. mentagrophyte* has been found to be abundant among school children in Abia state, Southeastern Nigeria [13]; among pupils in Oke-oyi, Kwara state in North Central Nigeria [10]; as well as Cross-River state, Nigeria [14]. The prevalence of *T. mentagrophyte* has been attributed to the perforating organ that the organism possesses. This facilitates the mechanical destruction of keratin and allows the faster growth of mycelia, which explains the dominance of this dermatophyte species [15]. Another peculiar finding in this study is the isolation of *M. gypseum* which we found in eight of the pupils. This particular dermatophyte has been said to be rarely isolated in Africa [15,16], although another study has shown that this fungi has been isolated in another region in Nigeria [6]. This shows that *M. gypseum* may be an emerging cause of dermatophyte infections in regions of the world where it was thought to be rare [16].

We found that tinea capitis is more prevalent in children between the ages of 4 and 7 years. This is similar to the findings by other studies, such as the study by George and Altraide (2008), which reported that most of the infected children were below the age of ten years [17]. These results support the suggestions that dermatophytosis, especially tinea capitis, is predominantly a pre-pubertal disease. Some of the explanations that have been accorded to this are that the fatty acids in the sebum produced at puberty may have some fungistatic properties, thereby preventing the infection of older children. For this reason, some studies even consider children in older age categories to be immune to tinea capitis [18]. Another factor that may support the higher prevalence of tinea capitis among younger children is the likelihood of poor hygiene in pre-pubertal stages, compared to older children who usually become more conscious of their hygiene practices when they reach their teenage years [12,19].

Regarding gender distribution, the infection was found to be more prevalent among male pupils [56.2%, (59/105)], compared to female pupils [43.8% (46/105)]. A plausible explanation could be that female pupils and their parents/guardians are more conscious of their appearance and hygiene practices than the males, as suggested in some previous studies [17,20]. We explored other important risk factors of dermatophytosis in this study. Poor hygiene is a significant risk factor. The poorer the hygiene, the higher the chances of contracting tinea capitis [11]. Our study showed a significant association between the infection and factors such as bathing with soap, irregular changing of uniform, playing with animals and sand, and the sharing of combs, caps and headscarves. This is in conformity with results from similar studies [7,11,17,21]. For these reasons, some schools have teachers conduct strict hygiene inspection, that is, teachers inspect pupils for the presence of lesions and enforce the frequent and proper washing of heads and handwashing practices. Lower prevalence of the infection has been observed in schools where the hygiene inspection intervention was practiced [22]. Socioeconomic status and living status (such as living with both parents) were not found to be statistically significant for tinea capitis in this study. However, we observed that about 48% and 62% of pupils from low- and middle-income households, respectively, have the infection.

The majority of the children live in houses where free-range animals are reared. We suspect that a thriving infection transmission link may exist between the pupils and their pets, or domesticated animals in their homes and neighborhoods. This is not very different from the study by Amen and Okolo (2004), which found a strong link between dermatophytosis and domestic animals [21]. A similar study also found that the overwhelming majority of the children who had tinea capitis had unlimited close contact with goats, sheep, and dogs that roam around and are not kept in closed pens [23]. The practice of rearing free-range and stray animals around the home and neighborhoods, which subsequently leads to the easy transmission of zoophilic tinea capitis, is further fostered in the cultural belief that the rearing of animals, especially free-range animals, keeps away evil from their owners [11]. In addition, routine veterinary care for animals is not particularly practiced. The gold standard treatment for tinea capitis is with the antifungal, griseofulvin. This treatment has contributed significantly to the reduction in the burden of this infection in most regions of the world [2]. However, the successful treatment of tinea capitis with griseofulvin has not been achieved in under-resourced regions of the world where the infection is endemic.

## 5. Conclusions

The findings from this study indicate a high prevalence of tinea capitis among children attending government-owned public primary schools in the Osogbo local government area, Osun state, Nigeria. The most prevalent dermatophytes found among the school children were *Tricophyton rubrum*, *Tricophyton mentagrophyte*, and *Microsporum canis*. There was a significant association between tinea capitis and the younger age group (4–7 years), certain unhygienic practices, and types of housing. There was a high prevalence of the infection among pupils who owned pets; this suggests a thriving animal–human transmission route of tinea capitis in the study area. However, this study did not carry out a detailed study to confirm the transmission route. We recommend intensive health promotion and education interventions to promote hygiene practices among school children, including the early detection and treatment of dermatophytosis among school children. School health care providers and teachers are important personnel in such programs. There is a need to promote routine veterinary checks for pets and domesticated animals, for the early detection and treatment of the infection.

## Figures and Tables

**Table 1:** Socio-demographic characteristics of the participants.

Socio-Demographic Characteristics	Frequency	Percent (%)
Age range (years)		
4–7	134	58
8–11	89	39
12–15	7	3
Gender		
Female	110	48
Male	120	52
Parents’ income status (salary per month)		
≤^1^#29,999 (low)	214	93
#3000–#74,999 (medium)	13	6
≥#75,000 (high)	3	1

^1^ #- Nigerian Naira.

**Table 2:** Prevalence of tinea capitis among pupils with clinical symptoms.

Results	Frequency	Percent (%)
Negative	10	9
Positive	105	91
Prevalence of Species		
*T. rubrum*	36	34
*T. mentagrophyte*	32	31
*M. canis*	19	18
*M. gypseum*	8	8
*E. flocussum*	6	6
*M. nauum*	3	3
*T. verucossum*	1	1

**Table 3:** Chi-square test of independence between the independent variables and the occurrence of tinea capitis. (**a**) Risk Factors Associated with the Occurrence of Tinea Capitis among All the Participants; (**b**) Risk Factors Associated with the Occurrence of Tinea Capitis among All the Participants, Based on Socio-Demographic Characteristics.

(**a**)			
The frequency of baths n, (%)			
	Negative (row%)	Positive (row%)	Total (column%)
Daily	83 (48)	89 (52)	172 (75)
More than once daily	42 (72)	16 (28)	58 (25)
Note.χ^2^ = 35.405, df = 1. * *p* < 0.001			
Use of soap for bath n, (%)			
	Negative (row%)	Positive (row%)	Total (column%)
Yes	124 (92)	11 (8)	135 (59)
No	1 (1)	94 (99)	95 (41)
Note. χ^2^ = 10.804, df = 1. * *p* < 0.001			
How often school uniform is changed n, (%)			
	Negative (row%)	Positive (row%)	Total (column%)
Daily	4 (100)	0 (0)	4 (2)
Once every week	52 (39)	82 (61)	134 (58)
Twice every week	63 (73)	23 (27)	86 (37)
More than twice weekly	6 (100)	0 (0)	6 (3)
Note. χ^2^ = 33.383, df = 3. * *p* < 0.001			
Playing habit n, (%)			
	Negative (row%)	Positive (row%)	Total (column%)
Play with animals	35 (34)	67 (66)	102 (44)
Play with sand	90 (70)	38 (30)	128 (56)
Note. χ^2^ = 29.649, df = 1. * *p* < 0.001			
(**b**)			
Age (years) n, (%)			
	Negative (row%)	Positive (row%)	Total (column%)
4–7	68 (51)	66 (49)	134 (58)
8–11	55 (62)	34 (38)	89 (39)
12–15	2 (29)	5 (71)	7 (3)
Note. χ^2^ = 4.566, df = 2. * *p* < 0.001			
Gender n, (%)			
	Negative (row%)	Positive (row%)	Total (column%)
Female	64 (58)	46 (42)	110 (48)
Male	61 (51)	59 (49)	120 (52)
Note. χ^2^ = 1.249, df = 1. * *p* = 0.264			
Socio-economic status n, (%)			
	Negative (row%)	Positive (row%)	Total (column%)
Low	120 (56)	94 (48)	214 (93)
Medium	5 (38)	8 (62)	13 (6)
High	0 (0)	3 (100)	3 (1)
Note. χ^2^ = 5.151, df = 2. * *p* = 0.076			
